# You Only Find What You Are Looking for: Concurrent Alcoholic Liver Cirrhosis and Undiagnosed Wilson’s Disease

**DOI:** 10.7759/cureus.17117

**Published:** 2021-08-12

**Authors:** Haya Omeish, Nada Hajjaj, Mohammad Abdulelah, Husam Bader

**Affiliations:** 1 Department of Internal Medicine, Royal Jordanian Medical Centre, Amman, JOR; 2 Department of Internal Medicine, The University of Jordan, Amman, JOR; 3 Department of Internal Medicine, Presbyterian Medical Center, Albuquerque, USA

**Keywords:** wilson’s disease, alcoholic liver disease, liver cirrhosis, ceruloplasmin, case report

## Abstract

Wilson’s disease (WD), a rare genetic disorder characterized by copper accumulation, leads to a spectrum of hepatic dysfunction including liver cirrhosis, fulminant liver failure, and chronic hepatitis. Its manifestations could involve musculoskeletal, hematologic, neuropsychiatric, or renal systems. We present the case of a 27-year-old female with a past medical history of alcohol use disorder who presented with acute confusion, worsening abdominal distension, bilateral lower limb edema, and jaundice.The initial presentation was concerning for acute alcoholic hepatitis and decompensated alcoholic cirrhosis attributed to ongoing heavy alcohol consumption. However, due to the patient’s young age, the severity of presentation, and the pattern of liver enzyme elevation, further workup was conducted to rule out concurrent pathologies. Viral, autoimmune, and metabolic workups were unrevealing. Subsequently, low ceruloplasmin levels and elevated urinary copper levels led to a diagnosis of WD with concomitant alcoholic liver disease.

The coexistence of WD and alcohol-associated liver disease (ALD) has not been well described in the literature. Laboratory testing including alkaline phosphatase (ALP), bilirubin, and serum aminotransferases provides the most rapid and accurate method for diagnosing ALD due to WD, given that the conventional screening tests such as ceruloplasmin are less sensitive and specific in identifying patients with acute liver disease secondary to WD.

## Introduction

Wilson’s disease (WD) is a rare autosomal recessive disorder [1]; it results from mutations in chromosome 13q14, in the region coding for the protein product ATP7B, which leads to defective incorporation of copper into apo-ceruloplasmin and the subsequent formation of holo-ceruloplasmin. This results in hampering the normal excretion of copper into the bile, leading to copper intoxication and deposition in various organs [2], thereby causing damage by direct oxidative stress leading to cell death [3]. As the liver is the primary organ for copper metabolism, hepatic changes are usually the earliest and most frequent manifestations in WD patients [4-6]. Liver cirrhosis is a common manifestation in WD patients [7]. Cirrhosis can be defined as the fibrotic replacement of the injured tissue by a collagenous scar and distortion of the hepatic architecture and formation of regenerative nodules [8]. The involvement of other organs reflects the accumulation of copper once the liver is saturated [9].

Cirrhosis is the final pathological pathway arising from a wide variety of chronic liver diseases. These include alcohol-associated liver disease (ALD), chronic hepatitis C virus (HCV) infection, chronic hepatitis B (HBV) infection, nonalcoholic fatty liver disease (NAFLD), inherited diseases such as hemochromatosis and WD, as well as autoimmune disorders such as primary biliary cirrhosis, primary sclerosing cholangitis, and autoimmune hepatitis [10]. Patients with liver cirrhosis usually remain asymptomatic for a long period of time until acute decompensation occurs. At that stage, patients experience complications of portal hypertension, which could include ascites, spontaneous bacterial peritonitis (SBP), hepatic encephalopathy (HE), hepatorenal syndrome, portopulmonary hypertension, or variceal bleeding [11]. HE is a severe complication and is associated with high mortality rates in cirrhotic patients [12]; it arises through portosystemic shunting, which allows ammonia and other neurotoxins to bypass the liver to enter the systemic circulation. The ultimate endpoint is cerebral edema as well as central nervous system inflammation, leading to complex cortical and subcortical dysfunction [13].

## Case presentation

A 27-year-old female with a past medical history pertinent for mild asthma, alcohol-use disorder (8-10 drinks/day), and generalized anxiety disorder presented to the emergency department after being found unconscious by her partner. The patient was unable to provide a coherent history upon presentation. Her partner reported that she had been experiencing unexplained low mood, generalized weakness, fatigue, intermittent confusion, and poor appetite for a few weeks. The aforementioned symptoms had exacerbated two days prior to the presentation. Further questioning revealed that the patient had been consuming large amounts of alcohol after losing her job due to the pandemic in early 2020.

The patient’s vital signs upon admission were not significantly alarming; her temperature was 36.1 °C, heart rate was 94 beats per minute, blood pressure was 101/66 mmHg, and her respiratory rate was 17 breaths/minute. The patient was noted to be confused with a Glasgow Coma Scale (GCS) of 12 (opened eyes to speech, confused and localized to pain). Further physical examination was notable for jaundice with icteric sclera, a distended protuberant abdomen with a positive fluid wave test, in addition to +1 bilateral lower limb edema. Asterixis could not be reliably evaluated.

Laboratory investigations revealed macrocytic anemia, mild thrombocytopenia, elevated liver enzymes with alanine transaminase (ALT) of 375 U/L and aspartate transaminase (AST) of 890 U/L, total bilirubin of 29 mg/dL, albumin of 2.1 g/dL, and blood ammonia concentration of 89 µmol/L. Detailed initial laboratory studies are listed in Table 1 and Table 2.

**Table 1:** Laboratory results upon admission INR: international normalized ratio

Laboratory studies	Values on admission	Reference range
Sodium (mmol/L)	130	135–145
Potassium (mmol/L)	3.1	3.6–5.0
Chloride (mmol/L)	110	98–109
Anion gap (AG) (mmol/L)	14	4–12
Blood urea nitrogen (mg/dL)	19	6–23
Creatinine (mg/dL)	0.37	0.67–1.17
Aspartate aminotransferase (U/L)	890	10–50
Alanine aminotransferase (U/L)	375	10–50
Alkaline phosphatase (U/L)	90	40–129
Bilirubin total (mg/dL), direct bilirubin (mg/dL), albumin (g/dL), INR	29.0, 17.3, 2.1, 1.6	0.2–1.3, 0.1–0.5, 3.5–5.5, 1–1.1
White blood cell count	8.8	4.22–10.33 × 10^9^/L
Hemoglobin (g/L)	11.9	13.3–16.2
Mean corpuscular volume (fL)	101	79.0–92.2
Platelet count	81	160–383 × 10^9^/L
Red cell distribution width (RDW)	22.8	<14%
Hematocrit	37	38.8–46.4
Glucose	58	<140 mg/dL (7.8 mmol/L)
Ammonia	89	15–45 u/dL (11–32 umol/L)
Lactic acid	3.5	0.5–2.2 mmol/L
Troponin	Negative	Negative
D-dimer	Negative	Negative

**Table 2:** Additional laboratory results ANA: antinuclear antibody; anti-LKM: anti-liver-kidney microsomal antibody; ASMA: anti-smooth muscle antibody

Laboratory studies	Results	Reference range
Ferritin (ng/mL)	Normal	30–400
Ceruloplasmin (mg/dL)	6	19–31
Copper, serum (μg/dL)	Not performed	63.7–140.12
Copper, urine 24 hours (μg/24 hours)	119	3–50
Hepatitis A virus antibody IgG	Non-reactive	Non-reactive
Hepatitis B surface antigen	Non-reactive	Non-reactive
Hepatitis B core antibody	Non-reactive	Non-reactive
Hepatitis B surface antibody	Non-reactive	Non-reactive
Hepatitis C antibody	Non-reactive	Non-reactive
ANA, anti-LKM, and ASMA	Not detected	Not detected

**Figure 1:**
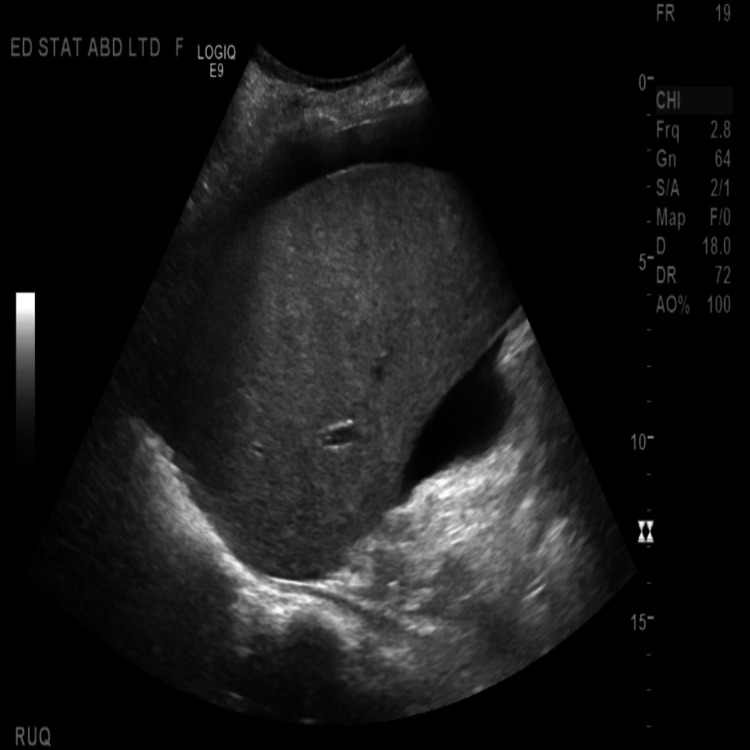
Abdominal US showing a diffusely enlarged liver with minimal nodularity US: ultrasound

**Figure 2:**
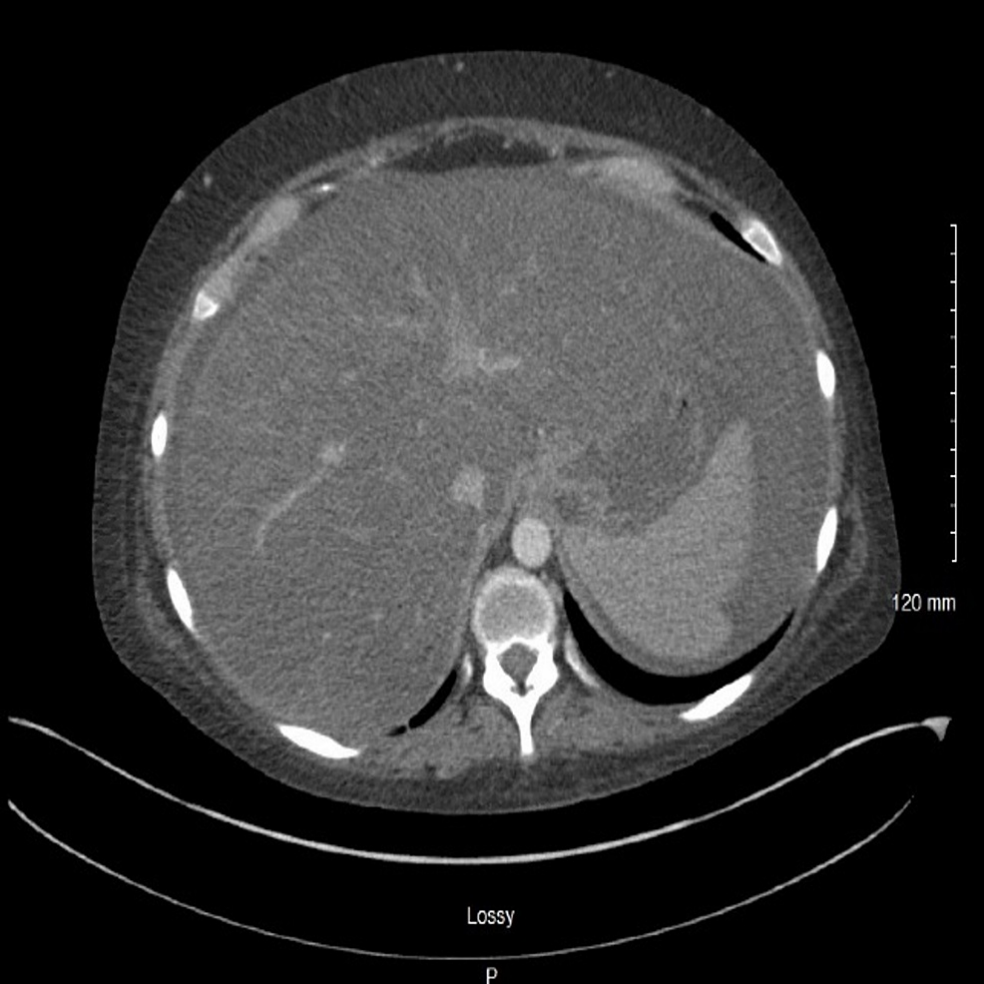
Abdominal CT demonstrates an enlarged liver, splenomegaly with a moderate volume of ascites, and cholelithiasis CT: computed tomography

The head CT scan was unremarkable. An abdominal ultrasound (US) demonstrated a diffusely echogenic and mildly enlarged liver with a minimally nodular surface, as well as ascites in all four quadrants. No intrahepatic or extrahepatic biliary obstruction was noted (Figure 1). Subsequent plain abdominal CT scan demonstrated an enlarged liver with diffuse heterogeneity of the liver parenchyma, without extra or intraductal dilation. A moderate volume of ascites was noted. Incidental cholelithiasis was also noted (Figure 2). Diagnostic paracentesis results (Table 3) were suggestive of portal hypertension without SBP [serum ascites albumin gradient (SAAG) of 1.3 and nucleated cell count of 89]. The fact that AST and ALT levels were more than 500 prompted further investigations into other potential causes of hepatic injury; an ischemic etiology was unlikely as the patient had a mean arterial pressure (MAP) above 65 on presentation and throughout hospitalization. Medication overdose and infectious, autoimmune, and genetic etiologies were also considered. Further workup included acetaminophen levels, hepatitis panel for HBV and HCV, ferritin level, antinuclear antibody (ANA), anti-liver-kidney microsome antibody (Anti-LKM), alpha 1 antitrypsin level, anti-smooth muscle antibodies (ASMA), and ceruloplasmin level. With the exception of ceruloplasmin level, all the other results were normal (Table 2). Ceruloplasmin was significantly low at 6 mg/dL. A confirmatory 24-hour urine copper level was high at 119 μg/24 hours. Liver biopsy was deferred in the acute setting given consistent clinical and biochemical pictures as well as the anticipated overall status improvement. The clinical picture was suggestive of either decompensated alcoholic liver cirrhosis or acute liver failure with an element of confounding alcohol intoxication. The elements suggesting liver cirrhosis included a history of heavy alcohol use, ascites with SAAG >1.3, and portal hypertension in the absence of portal vein thrombosis. However, abdominal CT scan and liver US were more suggestive of fatty infiltration of the liver rather than cirrhosis. Ideally, a liver biopsy would have helped assert the diagnosis but was perceived to be too invasive at that time. The patient was initially managed with lactulose and rifaximin to relieve symptoms of possible HE. Zinc was started as it interferes with the uptake of copper from the gastrointestinal tract [14]. Prednisone was also initiated given Maddrey's discriminant function of 52. Our liver transplant center was contacted; unfortunately, the patient did not meet the transplantation criteria at that time given the ongoing alcohol abuse and inadequate social support.

The patient’s mental status improved over the span of the next five days with complete resolution of confusion. The patient was scheduled for an ophthalmology clinic appointment to screen for Kayser-Fleischer rings while she was still in the hospital, which later confirmed the diagnosis. A referral to the hepatology clinic to further discuss alcohol abstinence, the usefulness of biopsy, and initiation of chelating agents was also scheduled. Given the patient’s deconditioning and high risk for clinical deterioration, she was discharged to a physical rehabilitation center with in-house alcohol recovery services.

**Table 3:** Paracentesis fluid analysis NRBC: nucleated red blood cells

Paracentesis fluid analysis	Results
Fluid source	Ascites
Fluid color	Yellow
Fluid appearance	Hazy
Nucleated cell count	89
Fluid neutrophils, %	7
Fluid lymphocytes, %	28
Fluid mononuclear, %	65
Fluid eosinophils, %	0
Fluid other cells, %	0
Fluid NRBC, %; albumin (g/dl)	0; 0.8
Paracentesis fluid culture	Negative

## Discussion

This case report discusses the challenges of an undiagnosed case of WD, especially in a setting where the medical history might lead to alternate differential diagnoses. Our patient’s initial presentation was concerning for acute liver failure, alcohol intoxication, or possible decompensated alcoholic liver cirrhosis. Alcohol could have been presumed to be the root cause; however, we were prompted to broaden our differential diagnoses and consider other potential etiologies concurrent with alcohol liver cirrhosis for three main reasons: 1) Patient’s age and the severity of the presentation. 2) The extent of AST/ALT elevation was higher than expected with alcohol alone (usual levels are <500) [15]. 3) The disproportionately mild elevation in ALP relative to the hyperbilirubinemia. According to the American Association for Study of Liver Diseases (AASLD), WD can be diagnosed without liver biopsy or molecular testing, by using the following criteria: serum ceruloplasmin of less than 0.20 g/l and raised 24-hour urine copper excretion that is greater than 100 μg/24 [16]. In the acute care setting, our patient was diagnosed with WD based on the aforementioned criteria without the need for biopsy or molecular testing. The presence of Kayser-Fleischer rings further confirmed the diagnosis later. It is prudent to mention that the standard laboratory tests for copper metabolism parameters including ceruloplasmin level and serum copper can vary widely in acute liver failure, which can be misleading. Higher diagnostic sensitivity and specificity are achieved when considering markers such as ALP and total bilirubin. A comparison of acute liver failure secondary to WD versus other conditions demonstrated that a ratio of ALP to total bilirubin <4.0 is 94% sensitive and 96% specific to diagnose WD. Additionally, the study found an AST to ALT ratio >2.2 is 94% sensitive and 86% specific to diagnose WD. Sensitivity and specificity for WD in the context of acute liver failure approached 100% when the two ratios were combined. Moreover, our patient’s biochemical profile was suggestive of WD, given ALP to total bilirubin ratio of 3.1 and AST to ALT ratio of 2.37 [17]. WD is a challenging diagnosis due to its rarity and wide range of clinical consequences. A high degree of suspicion is needed to not miss cases of WD, especially when coinciding with other common etiologies of liver failure and cirrhosis [18]. This diagnostic challenge has been well described in multiple cases, especially when two offending pathologies occur simultaneously. In some cases, WD can present in a fashion that is indistinguishable from autoimmune hepatitis, especially in young patients [19,20]. Given this resemblance, the AASLD’s current recommendations strongly encourage clinicians to carefully evaluate for WD in patients with autoimmune hepatitis who fail to respond rapidly and appropriately to corticosteroid treatment [7]. There are also a few reported cases in the literature describing viral infections, drug-induced hepatitis, or even NAFLD concomitantly coexisting with WD [7,20]. As ceruloplasmin and copper levels can be falsely normal in patients with acute liver failure secondary to WD, it is vital that clinicians pay attention to other biomarkers including ALP and total bilirubin. Even though the management of decompensated liver disease secondary to WD ideally requires liver transplantation, our patient did not qualify based on our center’s criteria. She will be followed up in the clinic and her sobriety and social support will be re-evaluated for reconsideration for liver transplantation.

## Conclusions

The case we presented highlights how the patient's presentation and biochemical markers prompted further investigations leading to the diagnosis of WD in the setting of obvious risk factors for more common etiologies of liver disease. This serves as a reminder that although alcohol and NAFLD are the most common causes of liver disease, clinicians should still maintain a high level of suspicion for concurrent liver pathologies in patients presenting with AST/ALT levels higher than 500 and patients younger than 40 years of age. This practice can ensure optimal treatment and avoid delayed diagnoses.
